# Structural Characterization of Sulfated Polysaccharide Isolated From Red Algae (*Gelidium crinale*) and Antioxidant and Anti-Inflammatory Effects in Macrophage Cells

**DOI:** 10.3389/fbioe.2021.794818

**Published:** 2021-11-18

**Authors:** Yu Pei, Shengtao Yang, Zhenbang Xiao, Chunxia Zhou, Pengzhi Hong, Zhong-Ji Qian

**Affiliations:** ^1^ College of Food Science and Technology, School of Chemistry and Environment, Shenzhen Institute of Guangdong Ocean University, Zhanjiang, China; ^2^ Southern Marine Science and Engineering Guangdong Laboratory, Zhanjiang, China

**Keywords:** Gelidium crinale, sulfated polysaccharide, structural characterization, anti-oxidation, anti-inflammation

## Abstract

*Gelidium crinale*, the red algae belonging to Geliaceae *Gelidium*, is a traditional edible and industrial alga in China. A sulfated polysaccharide (GNP) is successfully separated from *Gelidium crinale* by acid extraction and two-step column chromatography. Chemical analysis showed that the molecular weight of GNP was 25.8 kDa and the monosaccharide composition had the highest galactose content and confirmed the presence and content (16.5%) of sulfate by Fourier transform infrared spectroscopy (FT-IR) spectrometry as well as barium chloride-gelatin methods. In addition, the effect of GNP on lipopolysaccharide (LPS)-induced oxidative stress and inflammation in macrophages was also evaluated. The research results showed that GNP had fairly strong scavenging activities on 2,2′-azino-bis(3-ethylbenzothiazoline-6-sulfonic acid) (ABTS) radical, hydroxyl radical, and 1,1-diphenyl-2-picrylhydrazyl (DPPH) radical and had Fe^2+^-chelating ability in a dose-dependent manner. At the same time, it significantly inhibits the expression of inducible nitric oxide synthase (iNOS) and cyclooxygenase-2 (COX-2) and the production of pro-inflammatory cytokines in RAW 264.7 cells induced by LPS through blocking the mitogen-activated protein kinase (MAPK)/nuclear factor kappa beta (NF-κB) signaling pathway. These results indicate that GNP may be a latent component anti-inflammation in pharmaceutical and functional food industries.

## Introduction

Seaweeds are a natural source which not only contain a variety of essential nutrients but also meet the needs of therapeutic, pharmaceutical, and nutritional fields. Generally speaking, they are divided into three groups, namely, brown algae (*Phaeophyceae*), green algae (*Chlorophyceae*), and red algae (*Rhodophyceae*). According to reports, it has a variety of important biologically active compounds, such as lipids, polysaccharides, polyphenols, anthraquinones, steroids, flavonoids, alkaloids, triterpenoids, and cardiac glycosides (Cao et al., 2020). Among them, red algae had been widely used in multiple applications like agriculture, food, biomedical, and cosmetics. The red algal polysaccharides usually consist of carrageenan cellulose, starch, xylan, and porphyrin.

*Gelidium* species, the red algae, have been traditionally used in the phycocolloid industry for agar–agar production. It has been widely used in many countries. Among them, *Gelidium amansii* (GA) is a kind of widely widespread edible red algae and harvested in the Asian countries, including China, Korea, Japan, Thailand, and Singapore (Kang et al., 2016). Several studies have reported that GA has multiple biological activities, such as enhancing immune activities, preventing obesity caused by diet, anti-inflammatory effects, and improving lipid metabolism, among others (Wang et al., 2013; Liu et al., 2017; Yang et al., 2017) *Gelidium crinale* (GC), also known as Ma Mao (Shandong Province), Gou Mao Cai (Guangdong Province), and Yan Yi (Zhejiang Province), is a traditional edible marine economic red alga of *Gelidium* in China. Compared with GA by GC, it is clear that the morphology and size of algae are different, but the biological activity of components has not been studied. Therefore, the research is to extract sulfated polysaccharides from GC and evaluate their structure characterization and anti-inflammatory effects, as well as provide an experimental basis for the high-value utilization of GC.

Sulfated polysaccharides represent an important class of glycans. These polysaccharides are often endowed with high bioactivity related to their sulfate functional groups, which can interact with many positively charged biological macromolecules. In recent years, sulfated polysaccharides isolated from seaweeds (red algae and brown algae) have attracted more and more attention. Moreover, the sulfated polysaccharides in algae have anti-inflammatory, antioxidant, and other pharmacological activities (de Sousa Oliveira Vanderlei et al., 2011; Li et al., 2017; Lima de Castro et al., 2018) and can be used in nutrition and healthcare, pharmaceutical, and cosmetic industries. Moreover, inflammation is an important biological process for protecting the human body from diverse hazardous stimuli, such as infection, injury, and irritation (Kim et al., 2018). The characteristic of inflammation is that leukocytes migrate from blood to tissues and circulate in the tissues through proliferation, which relates to a range of adhesion processes between resident leukocytes and vascular endothelia. Choric inflammation and prolonged inflammation may be harmful and can lead to many diseases, including neurodegenerative diseases, fever, atherosclerosis, and even cancer (Ryu et al., 2017). Lipopolysaccharides, found in the outer membranes of Gram-negative bacteria, can activate a series of signaling pathways related to inflammation, such as NF-κB and MAPK pathways (Guha and Mackman, 2001). The activation of NF-κB promotes the expression of genes related to inflammation, including iNOS, COX-2, and pro-inflammatory cytokines (interleukin 6 (IL-6), interleukin-1β (IL-1β), and tumor necrosis factor-α (TNF-α)) (Baeuerle and Baichwal, 1997). In addition, the production of inflammation is also related to oxidative stress, and excessive reactive oxygen species (ROS) in cells can be considered as one of the causes of oxidative stress. The ROS molecule has high activity and plays a significant role in cell function. It can induce cells to secrete inflammatory factors and lead to inflammation. Overexpression of ROS can cause inflammation and promote the expression of inflammatory factors. Nitric oxide (NO) is also involved in the oxidation reaction caused by ROS, which promotes inflammatory response. Moreover, ROS can act as a second messenger of intracellular signal transduction and regulate iNOS, COX-2, and the expression of pro-inflammatory cytokines through MAPK/NF-κB activation; thus, inhibiting the level of ROS may be an anti-inflammatory method (Cobourne-Duval et al., 2016). Therefore, proper adjustment of the expression of inflammatory factors may reduce the adverse reactions of inflammation, thereby preventing the occurrence of inflammation-related diseases (Hou et al., 2020).

In the study, we first reported sulfated polysaccharides (GNP) isolated from *Gelidium crinale* (Naozhou Island Sea, Zhanjiang City). The structure of the polysaccharides was investigated through chemical analysis, Fourier transformation infrared spectroscopy (FT-IR), high-performance liquid chromatography (HPLC), and gel permeation chromatography system (GPC). The antioxidant activity of the obtained polysaccharides (including ABTS radical scavenging capacity, ferrous ion (Fe^2+^) chelating capacity, DPPH radical scavenging capacity, and hydroxyl radical scavenging capacity) was determined. In addition, the anti-inflammatory effects *in vitro* were evaluated, and the signal pathway was discussed in RAW 264.7 macrophages cell.

## Materials and methods

### Materials and Chemicals

Fresh *Gelidium crinale* were collected from about 1-m depth of Naozhou Island Sea, Zhanjiang City, Guangdong Province, in summer 2020. Algae were identified through the morphological characters of the herbarium and the appraisal scheme of the Prof. Zhang C (Guangdong Ocean University).

The standards (fucose, galactose, glucuronic acid, rhamnose, arabinose, ribose, xylose, glucose, and aminogalactose) were provided by Sigma-Aldrich (Sigma Chemicals, St. Louis, MO, USA). The bicinchoninic acid (BCA) assay kit and all cell culture chemicals were provided by Thermo Fisher Scientific, Inc. (Waltham, MA, USA). LPS, 2,7-dichlorodihydrofluorescein diacetate (DCFH-DA), 3-(4,5-dimethylthiazol-2-yl)-2,5-diphenyltetrazolium bromide (MTT), and dimethyl sulfoxide (DMSO) were purchased from Sigma-Aldrich (St. Louis, MO, USA). Mouse polyclonal antibodies, including p65 (sc-8008), p-p65 (sc-136548), IκBα (sc-1643), p-IκBα (sc-8404), p-JNK (sc-6254), JNK (sc-7345), p-p38 (sc-166182), and p-ERK (sc-81492); rabbit polyclonal antibodies (p38, (sc-535); ERK (sc-94)); and secondary antibodies, such as goat anti-rabbit IgG-HRP (sc-2004), and goat anti-mouse IgG-HRP (sc-2005) were provided by Santa Cruz Biotechnology (Santa Cruz, CA, USA).

### Preparation of Sulfated Polysaccharide from *Gelidium Crinal*

Sulfated polysaccharide was extracted using the method of Sun et al. (2018). *Gelidium crinale* (500 g) was extracted twice with 90% ethanol (W/V = 1:8) to remove pigments, lipids, and other impurities, and it was dried at 45°C and extracted with 0.1 M HCl (W/V = 1:8) for 8 h; the extraction solution was then neutralized. After centrifugation, the supernatant was condensed to one-fourth volume by a rotary evaporator at 50°C and was sedimented with 80% ethanol at 4°C overnight. The precipitate was taken after centrifugation, then redissolved with distilled water; the protein in the solution was removed with Sevag reagent, and the solution was concentrated and dialyzed.

In the second step of extraction, the solution was filtered through the Sepharose CL-6B column (2.5 × 60 cm), and the elution phase was 0.1 mol/l NaCl. Finally, the fractions were gathered and then freeze-dried to obtain the *Gelidium crinale* polysaccharide designated as native GNP.

### Chemical Composition Determination

The total sugar content was determined according to the phenol-sulfuric acid method (Nair and Vaidyanathan, 1964). The content of reducing sugar was determined by the 3,5-dinitrosalicylic acid (DNS) method. The sulfate content was determined by the barium chloride-gelatin method (Dodgson and Price, 1962).

### Monosaccharide Composition Determination

PMP (1-phenyl-3-methyl-5-pyrazolone) pre-column derivatization combined with HPLC was used to determine the monosaccharide composition of GNP. Firstly, GNP (10 mg) was hydrolyzed by trifluoroacetic acid solution for 4 h at 110°C and cooled to ambient temperature. Then, methanol (1 ml) was added and dried with nitrogen three to four times. One milliliter of NaOH (0.3 mol/l) solution was added to fully dissolve the residue, which is a polysaccharide hydrolysate, and derivatized after a certain dilution. Four hundred microliters of mixed monosaccharide standard solution or polysaccharide hydrolysate was taken respectively in a 5-ml test tube with a stopper, in which 400 μl of PMP methanol solution was added to mix and reacted in a 70°C water bath for 2 h. It was then cooled to room temperature, HCl was added to adjust the pH to 7, and the solution volume was diluted to 1 ml with water. Chloroform was added, let to stand as well as the organic solution to be discarded, and then extracted twice. The water phase was analyzed by HPLC after using a 0.45-μm microporous filter. Then, the mixture was further passed through an HPLC instrument equipped with a UV detector at 30°C column temperature and an Agilent Eclipse XDB-C18 column (250 × 4.6 mm, 5 μm) for detection. The mobile phase is phosphate buffer (pH = 6.6) and acetonitrile (Cao et al., 2019).

### Relative Molecular Weight Determination

The molecular weight was determined by the GPC system, which has a Waters 515 refractive index detector and a Shodex SBOHPAK-806-803 chromatographic column, and the column temperature is 40°C. The sample (500 μl) was injected, and the flow rate was set to 1 ml/min. The mobile phase was ultrapure water (0.02% sodium azide, pH = 6). The time and logarithm of molecular weight were used as the abscissa and ordinate, respectively (Cui et al., 2019). The Jiangshen workstation (produced by Dalian Jiangshen Chromatography Software Co., Ltd.) for data processing to obtain the weight average molecular weight (Mw), number average molecular weight (Mn), and molecular weight distribution Mw/Mn.

### Fourier Transform Infrared Spectroscopy Analysis

Potassium bromide was first ground with an agate mortar, then passed through a mesh screen with an aperture of 0.147 mm and dried under an infrared light for 4 h. Potassium bromide was pressed into a translucent sheet as a blank, and the sample was mixed with potassium bromide to make a pressed sheet, and the scanning was performed in the range of 4,000–400 cm^−1^ (Jia et al., 2015).

### *In vitro* Antioxidant Activities

#### ABTS Radical Scavenging Capacity Assay

The ABTS radical scavenging capacity was measured according to the method of Wang et al. (2021). Potassium persulfate at 2.45 mM and ABTS at 7 mM were mixed in equal volumes and kept protected from light for 16 h at room temperature to prepare ABTS radicals. ABTS free radicals were diluted in phosphate-buffered saline (PBS) to an absorbance of 0.70 ± 0.02 at 734 nm. Then, 2.5 ml of ABTS radical and 0.5 ml of different concentrations of GNP were mixed and reacted for 25 min at room temperature. Last, the absorbance of the mixture was measured at 734 nm and calculated according to the following formula, where Ai is the absorbance of GNP mixed with the reaction solution; A0 is the absorbance of the mixture solution without sample; and Aj is the absorbance of GNP.

$$\mathrm{ABTS}\;\mathrm{radical}\;\mathrm{scavenging}\;\mathrm{capacity}\;\left(\text{\%}\right)=\frac{\left(A0-\mathrm{Ai}+\mathrm{Aj}\right)}{A0}\times \mathrm{100}\text{\%}$$

#### Ferrous Ion (Fe^2+^) Chelating Capacity Assay

The ferrous ion chelating potency was measured according to the method of Sun et al. (2018). One milliliter of GNP with different concentrations was mixed with 0.2 ml of ferrozine (5 mM) and 0.1 ml of ferrous chloride (2 mM) and let to stand at room temperature for 15 min. Last, the absorbance of the mixture was measured at 562 nm and calculated according to the following formula.

$$\mathrm{Ferrous}\;\mathrm{ion}\;\mathrm{chelating}\;\mathrm{rate}\left(\text{\%}\right)=\frac{\left(A0-\mathrm{Ai}+\mathrm{Aj}\right)}{A0}\times \mathrm{100}\text{\%}$$

#### DPPH Radical Scavenging Capacity Assay

The DPPH radical scavenging capacity was measured according to the method of Jia et al. (2015). In short, 4 ml of DPPH (0.5 mmol/l) and 2 ml different concentrations of GNP were mixed and let to stand for 30 min. Last, the absorbance of the mixture was measured at 517 nm and calculated according to the following formula.

$$\mathrm{DPPH}\;\mathrm{radical}\;\mathrm{scavenging}\;\mathrm{capacity}\left(\text{\%}\right)=\frac{\left(A0-\mathrm{Ai}+\mathrm{Aj}\right)}{A0}\times \mathrm{100}\text{\%}$$

#### Hydroxyl Radical Scavenging Capacity Assay

The hydroxyl radical scavenging ability was measured according to the method of Wang et al. (2021). In short, 2 ml of different concentrations of GNP, 2 ml H_2_O_2_ (9 mM), and 2 ml FeSO_4_ (9 mM) were mixed and reacted at 25°C for 10 min. Then, 2 ml salicylic acid (9 mmol/l) was added and reacted for 30 min. Last, the absorbance of the mixture was measured at 540 nm and calculated according to the following formula.

$$\mathrm{Hydroxyl}\;\mathrm{radical}\;\mathrm{scavenging}\;\mathrm{capacity}\left(\text{\%}\right)=\frac{\left(A0-\mathrm{Ai}+\mathrm{Aj}\right)}{A0}\times \mathrm{100}\text{\%}$$

### Cell Cultures and Cell Viability Assay

RAW 264.7 cells were provided by Fudan IBS Cell Resource Center. Cells were plated in DMEM and contained 10% fetal bovine serum (FBS) and 1% penicillin–streptomycin in 5% CO_2_ at 37°C. RAW 264.7 cells were seeded into a 96-well plate and incubated at 37°C for 24 h, and then GNP (1, 10, 50, 100, 200, 500, and 1,000 μg/ml) was added to the wells. Then, 100 μl (0.5 mg/ml) MTT was added to each well and left for 4 h. After cultivation, the supernatants were removed and the dark blue crystals were dissolved with dimethyl sulfoxide. Also, the absorbance of the mixture was measured at 540 nm with a microplate reader (Wang et al., 2021).

### NO Production

The amount of NO released was measured by the Griess method. Briefly, RAW 264.7 cells were seeded into a 96-well plate at a concentration of 5 × 10^3^ cells/ml, and then cells were treated with GNP and LPS for 24 h. Fifty microliters of supernatant was collected and mixed with the NO detection kit. The absorbance was determined at 540 nm (Jia et al., 2020).

### Productions of Intracellular ROS Assess

RAW 264.7 cells were seeded into a 96-well plate, then cells were treated with GNP and LPS for 24 h, and then DCF (10 µM) was added into each well. After 30 min of incubation in the dark, the cells were washed with PBS. Subsequently, Hoechst 33342 (5 μg/ml) was added and incubated for 10 min. The fluorescence values of Hoechst 33342 and DCF were measured with a microplate reader. The excitation and emission wavelengths for DCF are 485 and 530 nm, respectively; those for Hoechst 33342 were 500 and 460 nm, respectively (Yu et al., 2016).

### ELISA Analysis of Cytokines

RAW 264.7 cells were treated with GNP and LPS for 24 h. According to the manufacturer’s instructions, the ELISA kit was used to measure the concentrations of TNF-α and IL-6 in the supernatant (Xiao et al., 2020).

### Western Blot

RAW 264.7 cells were seeded into a six-well plate at a concentration of 5 × 10^6^ cells/ml and cultured for 24 h. The old medium was aspirated and discarded and then treated with GNP for 2 h, then LPS was added for a 24-h incubation. The protein content was determined with the BCA protein quantification kit and separated by electrophoresis. Then, it was shifted to NC membranes. The membrane is sealed with 5% skimmed milk for 2 h and incubated with primary antibody (dilution ratio 1: 500) at 4°C. The secondary antibody (dilution ratio of 1: 2,000) was incubated for 2 h and then washed with TBST three times. Protein expression levels were detected by using enhanced chemiluminescence substrates (Yang et al., 2020).

### Statistical Analysis

All results represent the average of three independent experiments. GraphPad Prism 8 (GraphPad Prism Software Inc., La Jolla, CA, USA) and ImageJ (Version 1.46r, NIH, Bethesda, MD, USA) were used for data analyses, and statistical analyses between different groups were performed by t-tests or one-way ANOVA. *p* < 0.05 was judged to be statistically significant and was highlighted with asterisks.

## Results and discussion

### Chemical Composition and Monosaccharide Composition of GNP

**FIGURE 1:**
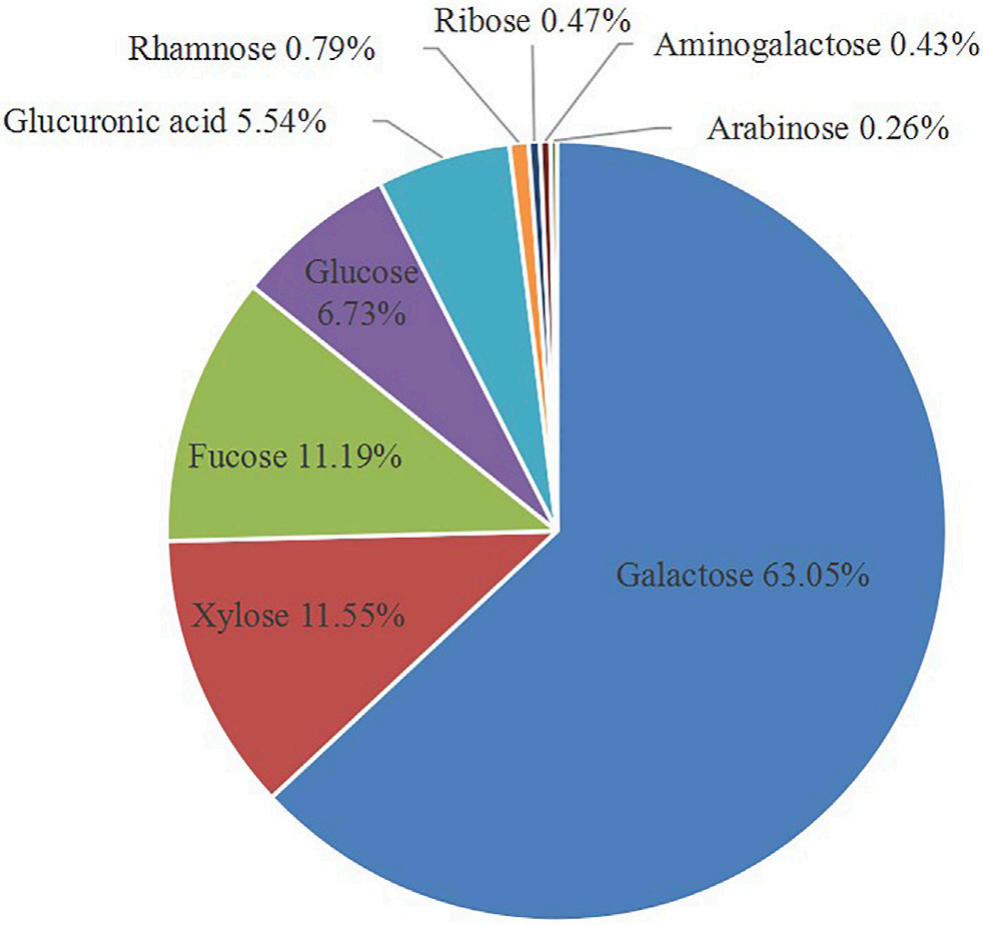
Monosaccharide compositions of GNP were determined by the method of PMP (1-phenyl-3-methyl-5-pyrazolone) pre-column derivatization combined with high-performance liquid chromatography (HPLC).

Acid extraction is a commonly used method for extracting polysaccharides, which shows a relatively high sulfate content and improves the biological activity. The acid extraction of GNP was processed with treatment of 0.1 M HCl. The total yield of GNP by several times of acid extraction process was about 3.2% on the basis of lyophilized dry weight. Then, the impurities were removed and the total sugar content was determined, and the content was increased to 75.78% (date not show). In addition, as shown in Table 1, the reducing sugar content was 8.42%, and the sulfate group content was 16.50%. PMP pre-column derivatization and HPLC analysis of the monosaccharide composition of GNP were performed. As shown in Figure 1, the GNP was mainly composed of galactose (65.05%), xylose (11.55%), fucose (11.19%), glucose (6.73%), glucuronic acid (5.54%), rhamnose (0.79%), ribose (0.47%), amino galactose (0.43%), and arabinose (0.26%). Among them, galactose has the highest content, followed by fucose and xylose. Galactose accounts for more than half of all monosaccharides. Cui et al. (2019) used the GC-MS analysis method to analyze the monosaccharide composition of sulfated polysaccharide from the red seaweed *Gelidium pacificum*. Research indicated that the main monosaccharide composition of GPOP-1 was galactose (59.7%), xylose (7.1%), and galacturonic acid (19.76%); it was determined that the sulfate group content was 8.80%. Moreover, Yang et al. (2019) analyzed the monosaccharide composition of polysaccharide from the red algae GA, and the results showed that the main monosaccharide composition of GHE was galactose (86.0%), fucose (8.3%), and xylose (1.1%). GNP obviously accords with the characteristics of the *Gelidium amansii* polysaccharide. Moreover, the sulfuric acid group content of GNP is relatively high. Many studies have proved that the higher the sulfate content, the better the biological activity of polysaccharides (Hou et al., 2020).

**TABLE 1:** The chemical properties and molecular weight of GNP.

Parameters	GNP
Chemical properties	
Reducing sugar content	8.42%
Sulfate content	16.50%
Molecular weight	
Weight-average molecular weight (Mw)	25.77 kDa
Number-average molecular weight (Mn)	13.39 kDa
Polydispersity (Mw/Mn)	1.92

Relative molecular weight of GNP.

As shown in Table 1, the molecular weight of GNP was analyzed using the GPC method to obtain several molecular weights, such as weight average molecular weight (Mw), number average molecular weight (Mn), and polydispersity (Mw/Mn). Research indicated that the Mn and Mw of GNP were 13.39 and 25.77 kDa, respectively, and the Mw/Mn was 1.92. Jia et al. (2015) indicated that the smaller molecular weight of the polysaccharide isolated and purified from R. minima root had a higher cancer-destroying activity. Furthermore, Sun et al. (2018) extracted polysaccharides from *Laminaria japonica* and found that polysaccharides with smaller molecular weight had a higher antioxidant activity. Meanwhile, Dou et al. (2019) discovered that the lower molecular weight blackberry polysaccharide had a stronger bile acid-binding ability and was more easily used by intestinal bacteria.

Bioactivities of GNP are related to its structure, including sulfate group content and relative molecular weight. Studies have shown that polysaccharides with smaller relative molecular weight and higher sulfate content had better biological activity (Hou et al., 2020; Mou et al., 2018). It is shown in Table 2 that the molecular weight of red algae polysaccharides ranges from tens to hundreds of kDa, and the sulfate group content is generally about 10%. However, GNP has a higher content of sulfate groups and a smaller molecular weight.

**TABLE 2:** Comparison of the sulfuric acid content and molecular weight of different types of red algae.

Raw material name	Sulfate content (%)	Molecular weight (kDa)	Physical map	References
*Gracilaria caudata*	—	250	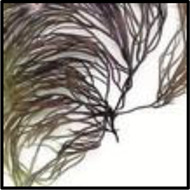	Barros et al. (2013)
*Gracilaria birdiae*	6.4	—	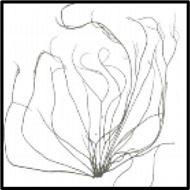	Maciel et al. (2008)
*Gloiopeltis tenax*	8.2	—	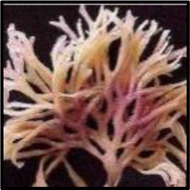	Lim and Ryu, (2009)
*Solieria filiformis*	—	28	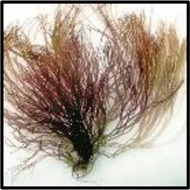	Chaves et al. (2018)
*Gloiopeltis furcata*	24.8	20.6	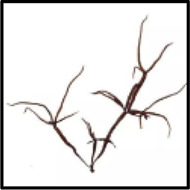	Hu et al. (2012)
*Gracilaria intermedia*	6.6	—	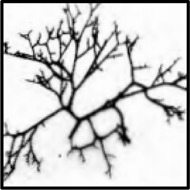	Lima de Castro et al. (2018)
*Gracilaria corticata*	—	43	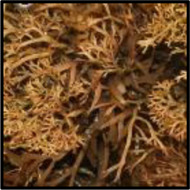	Seedevi et al. (2017)
*Gelidium pacificum Okamura*	8.8	28.81	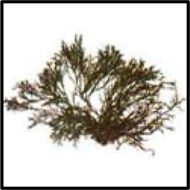	Cui et al. (2019)
*Gelidium amansii*	3.72–4.02	31.62–75.86	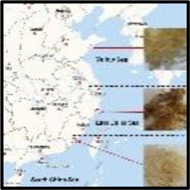	Yu et al. (2021)
*Gelidium crinale*	16.50	25.77	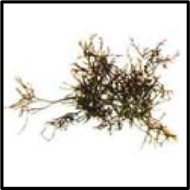	No reports

### FT-IR Spectral Analysis

**FIGURE 2:**
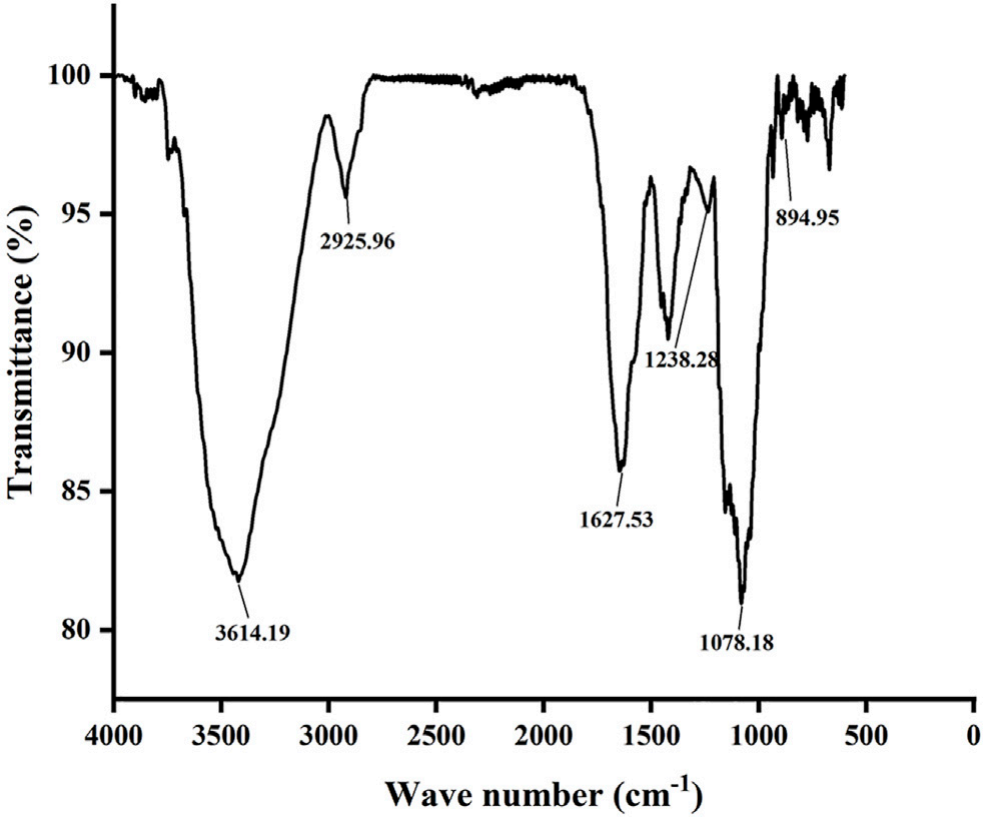
FT-IR spectroscopy of GNP.

It is shown in Figure 2 that there is an absorption peak at 3,400 cm^−1^, which is closely related to the O–H bond stretching of polysaccharides, indicating that there are power intra-molecular or intermolecular interactions between polysaccharide chains (Wang et al., 2021). The weak band at about 2,930 cm^−1^ is closely related to the C–H tensile vibration (Jiao et al., 2018). The absorption band in the range of 1,600–1,650 cm^−1^ is caused by C=O asymmetric stretching vibration, which proves that the polysaccharide contains uronic acid, which is an acidic polysaccharide (Shu et al., 2018). There is a stretching vibration of S=O at 1,200–1,250 cm^−1^, and S=O is the characteristic group of the sulfate group (Sanjeewa et al., 2018). The strong absorption band at approximately 1,000–1,200 cm^−1^ is closely related to the existence of C–O–H and C–O–C stretching vibrations, which are pyranose rings (Zhu et al., 2021). In addition, the characteristic absorption at 896 cm^−1^ indicates the presence of β configuration in polysaccharides (Bi et al., 2018). Therefore, these characteristics demonstrated that GNP is a sulfated polysaccharide.

### *In vitro* Antioxidant Activities

**FIGURE 3:**
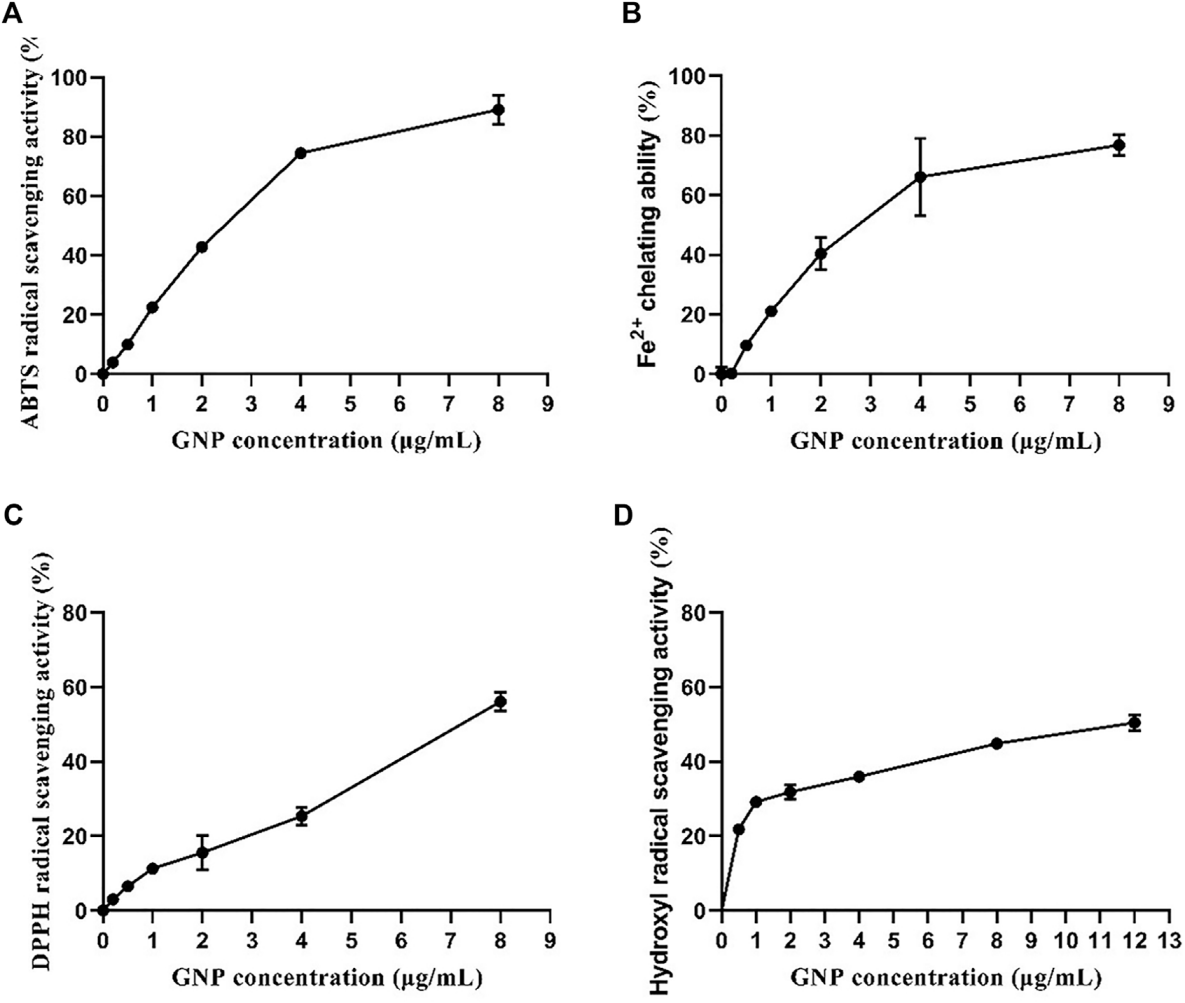
*In vitro* antioxidant activities of GNP. **(A)** ABTS radical scavenging activity, **(B)** Fe^2+^ chelating capacity, **(C)** DPPH radical scavenging activity, and **(D)** hydroxyl radical scavenging activity. Data are expressed as mean ± SD (n = 3).

The antioxidant activities of GNP estimated through four experiments are shown in Figures 3A–D. GNP exhibits extremely antioxidant activities in a concentration-dependent manner. The ABTS radical scavenging capacity, Fe^2+^ chelating ability, and DPPH radical scavenging capacity were 89.10%, 76.81%, and 56.11%, respectively, when the consistency of GNP was 8 mg/ml. The hydroxyl free radical scavenging ability was 50.43%, with the consistency of GNP of 12 mg/ml. The IC_50_ of its ABTS free radical scavenging ability, Fe^2+^ chelating ability, hydroxyl free radical scavenging ability, and DPPH free radical scavenging ability were 2.22, 2.69, 13.56, and 7.41 mg/ml, respectively. Many studies have reported that sulfated polysaccharides are good protective agents for antioxidant enzymes in cells. Thus, the sulfate content and molecular weight of sulfated polysaccharides will have a certain impact on the antioxidant activity (Andrew and Jayaraman, 2021). Sun et al. (2018) indicated that Laminaria japonica polysaccharides with a higher content of sulfuric acid groups and a smaller molecular weight have a strong ability to scavenge free radicals. Rozi et al. (2019) extracted polysaccharide from *Fritillaria pallidiflora* and believed that its hydroxyl radical scavenging activity was higher due to its lower molecular weight. Hence, GNP can be used as potential radical scavengers.

### Cell Viability Assay

**FIGURE 4:**
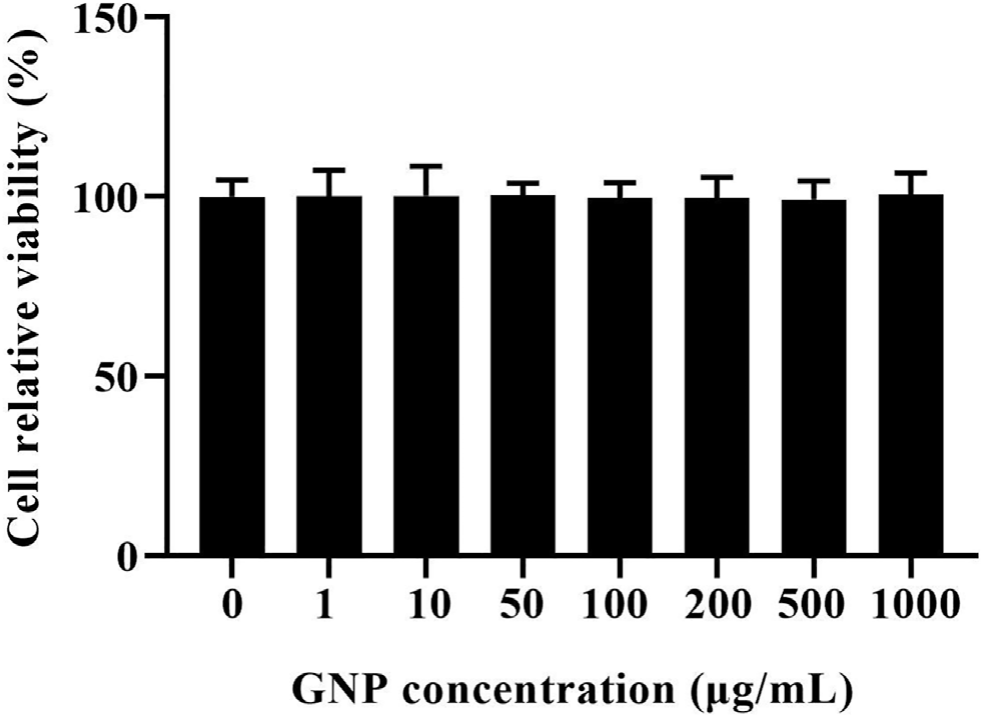
Effect of GNP on the viability of RAW 264.7 cells. Cells were treated with GNP (10, 50, 100, and 200 μg/ml) for 24 h. Cell viability was detected by MTT assay. Data are expressed as mean ± SD (n = 3).

In order to avoid the unnatural death of RAW 264.7 cells induced by GNP, the effect of GNP on the viability of RAW 264.7 cells was detected by MTT analysis (Du et al., 2018). It is shown in Figure 4 that compared with the blank group without GNP, the cell survival rate of the experimental group with GNP (1, 10, 50, 100, 200, 500, and 1,000 μg/ml) did not change significantly. It shows that the GNP has no poison effect on RAW 264.7 cells, and GNP at this concentration can be selected for subsequent experiments.

### Effects of GNP on NO, ROS, and Inflammatory Cytokine Production

**FIGURE 5:**
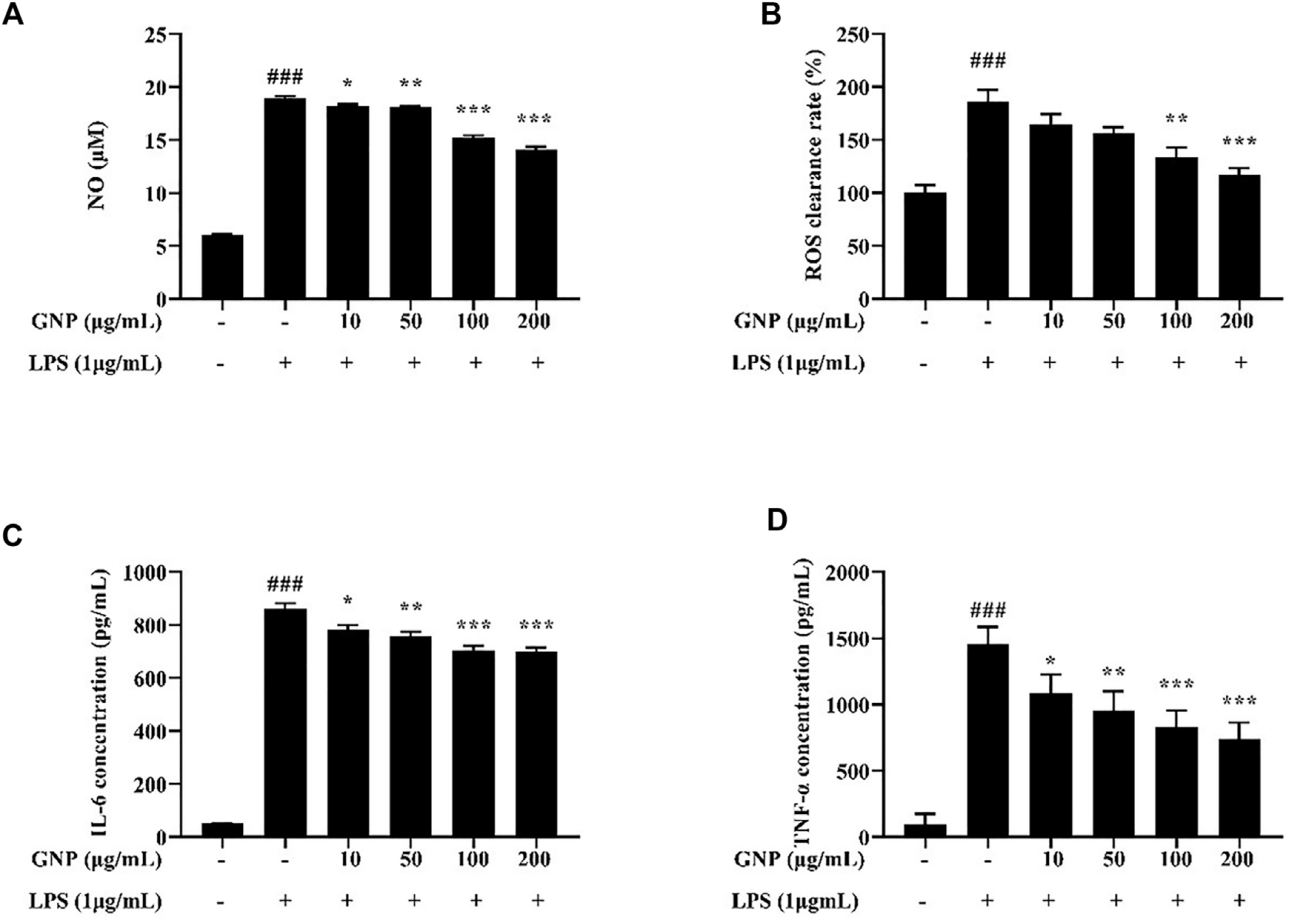
**(A)** Effect of GNP on NO levels in LPS-induced RAW 264.7 cells. **(B)** Effect of GNP on ROS levels in LPS-induced RAW 264.7 cells. **(C)** Effect of GNP on TNF-α levels in LPS-induced RAW 264.7 cells. **(D)** Effect of GNP on IL-6 levels in LPS-induced RAW 264.7 cells. Data are expressed as mean ± SD (n = 3). (# indicates the significant difference between the LPS group and the blank group, #*p* < 0.05, ##*p* < 0.01, ###*p* < 0.001; * indicates a significant difference between the experimental group and the LPS group, *p < 0.05, **p < 0.01, ***p < 0.001).

Macrophages are a type of immune cells that participate in the inflammatory responses. However, uncontrolled inflammation would lead to tissue damage and further diseases. Inflammation can lead to the secretion of inflammatory mediators (Rahmati et al., 2016; Hou et al., 2020). It is shown in Figures 5A–D that the levels of NO and ROS in RAW 264.7 cells treated with 1 μg/ml LPS for 24 h significantly increased. However, GNP can reduce the levels of NO and ROS in cells stimulated by LPS, indicating that GNP can relieve LPS-induced inflammation. Wang et al. (2020) reported that SNPS inhibited LPS-induced TNF-α and IL-6 protein and mRNA expression levels in RAW 264.7 cells. Moreover, Le et al. (2020) showed that MESP inhibits the levels of NO, TNF-α, IL-1β, IL-6, and IL-10 to reduce LPS-induced inflammatory response in macrophages. Meanwhile, Wang et al. (2021) found that CGP-BG inhibited the protein levels of NO, IL-1β, IL-6, and TNF-α by LPS induction. Therefore, it can be explained that GNP has anti-inflammation activity by inhibiting inflammatory mediators.

### Effects of GNP on iNOS and COX-2 Protein Expression

**FIGURE 6:**
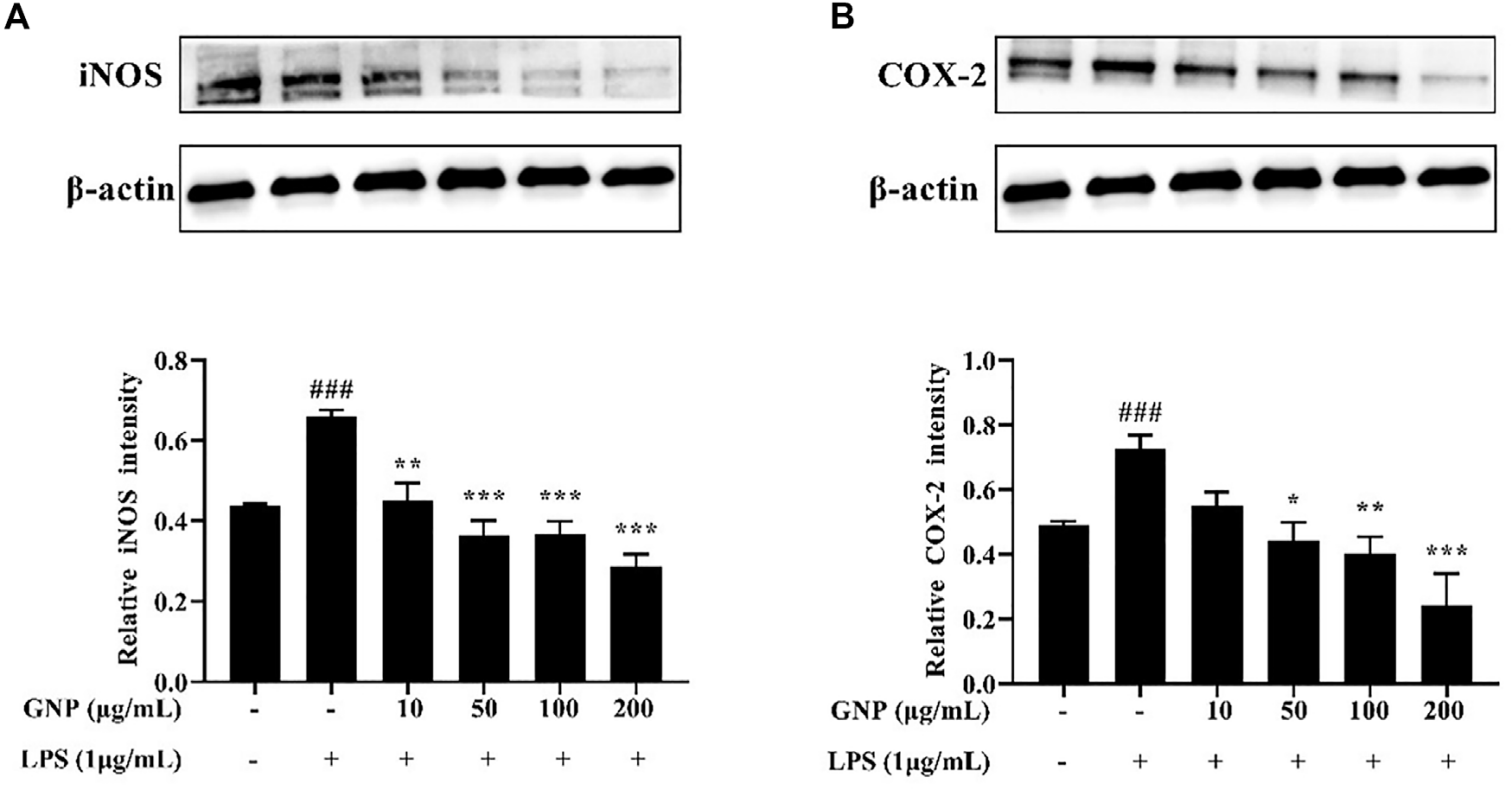
Effect of GNP on iNOS and COX-2 protein levels in LPS-induced RAW 264.7 cells. Cells were treated with GNP (10, 50, 100, and 200 μg/ml) for 2 h and then treated with LPS (1 μg/ml) for 24 h **(A)** iNOS protein expression and **(B)** COX-2 protein expression. Data are expressed as mean ± SD (n = 3). (# indicates the significant difference between the LPS group and the blank group, #*p* < 0.05, ##*p* < 0.01, ###*p* < 0.001; * indicates a significant difference between the experimental group and the LPS group, *p < 0.05, **p < 0.01, ***p < 0.001).

iNOS can induce the production of NO and is an indicator to identify whether inflammation occurs; COX-2 is also involved in inflammation. They all catalyze the production of a large number of pro-inflammatory mediators. Therefore, inhibiting its activity can effectively reduce the degree of inflammation (Du et al., 2018; Zhu et al., 2018; Hou et al., 2020). Sanjeewa et al. (2018) reported that sulfated polysaccharide from *Sargassum horneri* dose-dependently inhibited the expression of COX-2 and iNOS. Meanwhile, Wu et al. (2016) reported that sulfated polysaccharide from the brown alga *Sargassum cristaefolium* can inhibit the expression of iNOS. It is shown in Figures 6A,B that the protein levels of COX-2 and iNOS in cells increased significantly after LPS treatment in cells, but their protein levels decreased after GNP treatment. GNP can inhibit the expression of iNOS and COX-2 in cells induced by LPS. It can be seen that GNP can inhibit the expression of iNOS and COX-2 in cells to play an anti-inflammatory effect.

### The Effect of GNP on the NF-kB Signaling Pathway

**FIGURE 7:**
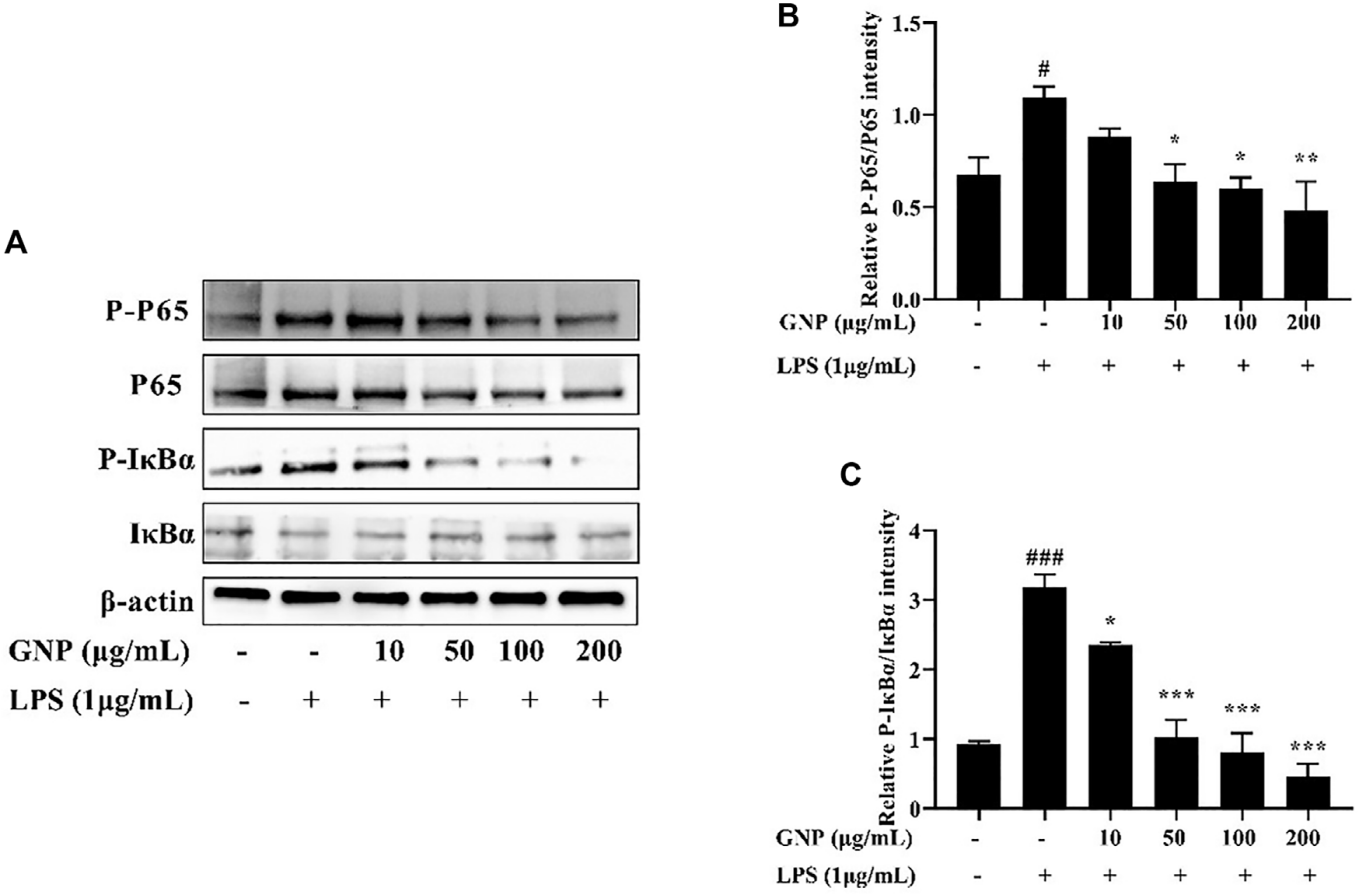
Effect of GNP on the LPS-induced activation of the NF-κB transcription factor in RAW 264.7 cells. **(A)** The phosphorylation levels of p65, p-p65, IκBα, and p-IκBα proteins in RAW 264.7 cells. Cells were treated with GNP (10, 50, 100, 200 μg/ml) for 2 h and then treated with LPS (1 μg/ml) for 24 h. **(B)** The ratios of p-p65/p65. **(C)** The ratios of p-IκBα/IκBα. Data are expressed as mean ± SD (n = 3). (# indicates the significant difference between the LPS group and the blank group, #*p* < 0.05, ##*p* < 0.01, ###*p* < 0.001; * indicates a significant difference between the experimental group and the LPS group, *p < 0.05, **p < 0.01, ***p < 0.001).

NF-κB participates in the transactivation of various genes related to the regulation of immune and inflammatory responses. It is the most important transcription factor and consists of homodimers or heterodimers of Rel proteins (Du et al., 2015). In normal cells, it binds to IκB and locates in the cytoplasm, thereby inhibiting its entry into the nucleus. Then, phosphorylation of IκB causes NF-κB to be activated to enter the nucleus, thereby starting to produce various mediators (Levy-Ontman et al., 2011; Wu et al., 2016). As shown in Figures 7A–C, the gray levels of the bands were analyzed in each group. After LPS treatment, the protein expression levels of p-p65 and p-IκBα in RAW 264.7 cells increased significantly, while after GNP treatment, their protein expression decreased. This indicates that GNP can block NF-kB from entering the nucleus by inhibiting the phosphorylation of p65 and IκBα.

### The Effect of GNP on the MAPK Signaling Pathway

**FIGURE 8:**
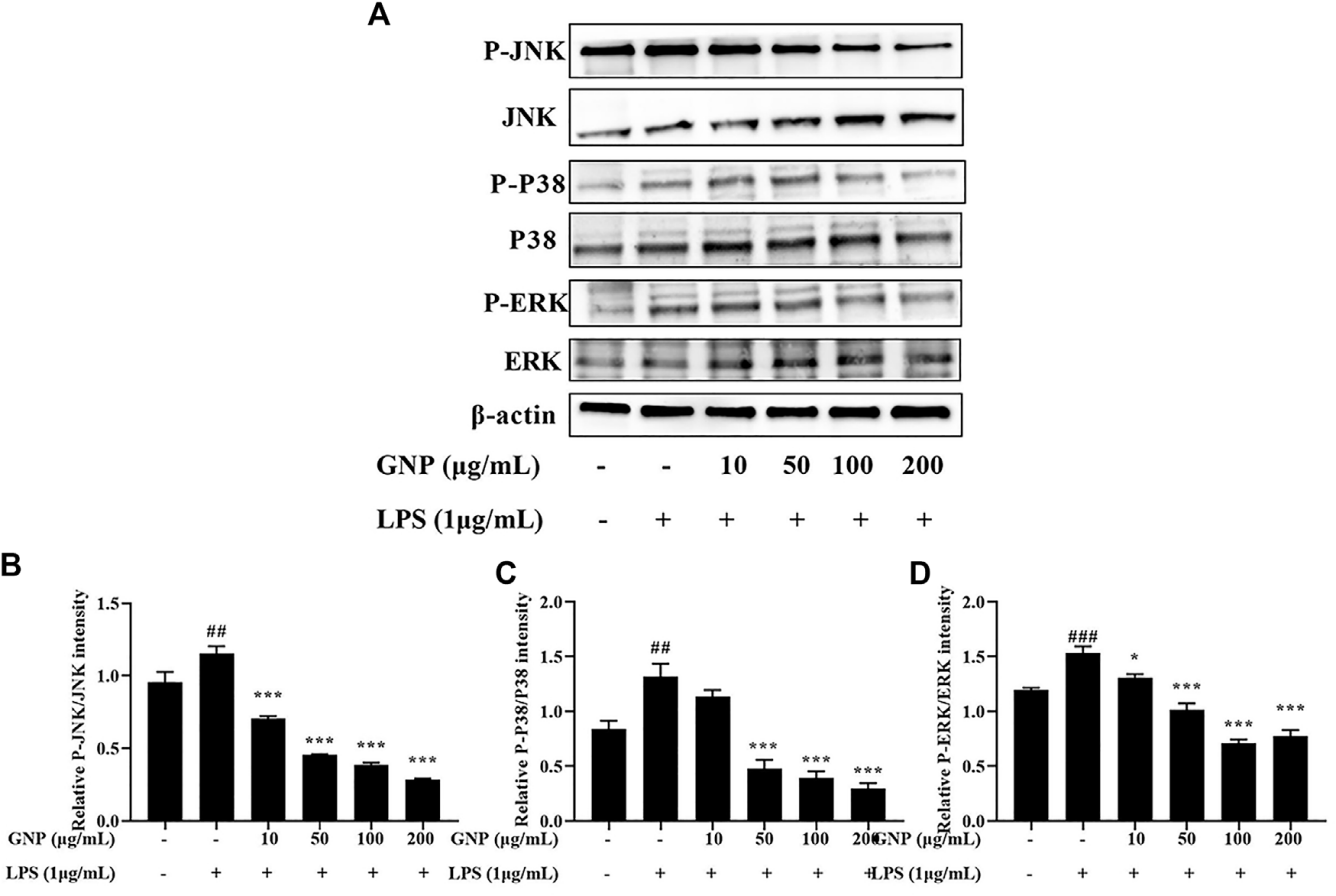
Effect of GNP on the LPS-induced phosphorylation of MAPKs in RAW 264.7 cells. **(A)** The phosphorylation levels of JNK, p-JNK, p38, p-p38, ERK, and p-ERK proteins in RAW 264.7 cells. Cells were treated with GNP (10, 50, 100, and 200 μg/ml) for 2 h and then treated with LPS (1 μg/ml) for 24 h. **(B)** The ratios of p-JNK/JNK. **(C)** The ratios of p-p38/p38. **(D)** The ratios of p-ERK/ERK were calculated. Data are expressed as mean ± SD (n = 3). (“#” indicates the significant difference between the LPS group and the blank group, #*p* < 0.05, ##*p* < 0.01, ###*p* < 0.001; “*” indicates a significant difference between the experimental group and the LPS group, *p < 0.05, **p < 0.01, ***p < 0.001).

Mitogen-activated protein kinase (MAPK), including extracellular signal-regulated kinase (ERK), c-Jun NH2-terminal kinase (JNK), and P38 participate in the mediation of cell growth, apoptosis, and proliferation (Arthur et al., 2013). MAPK is the upstream signaling molecule of NF-κB, and it also participates in the inflammatory response and regulates the expression of related genes (Paunovic and Harnett, 2013; Jia et al., 2020). This study evaluated the expression of ERK, JNK, and p38 by Western blotting. It is shown in Figures 8A–D that the protein expression levels of p-p38, p-JNK, and p-ERK in RAW 264.7 cells were increased after LPS induction. However, after GNP treatment, their protein levels decreased. This indicates that GNP can block the MAPK signaling pathway by restraining the phosphorylation of P38, JNK, and ERK.

From the structure–activity relationship, the GNP from *Gelidium crinale* has rich potential sulfate content (16.5%), galactose content (63.05%), and smaller molecular weight (25.8 kDa) compared with other red algae (*Gloiopeltis furcata*, *Gracilaria intermedia*, *Gelidium pacificum Okamura*, and *Gelidium amansii*, Table 2). In red algae, sulfate content, monosaccharide composition, and relative molecular weight are important factors for the activity level and intensity. Sulfate content might affect the binding of polysaccharides with the cell wall receptor, thereby affecting the production of NO, but the specific reason for it still unknown (Hou et al., 2020). A lot of research has shown that the lower molecular weight makes the spatial conformation of the polysaccharides easy to be combined with macrophage cells, especially polysaccharides with molecular weight of 10–200 kDa, which are the most active (Zhang et al., 2016; Ji et al., 2018). In addition, glucose, galactose, and mannose combine with other monosaccharides to produce polysaccharides with high activity, which are also important structure–activity groups in active polysaccharides (Zhang et al., 2016). Our results show that the structure and monosaccharide compositions of GNP are similar to other studies, which is completely consistent with the properties of red algal polysaccharides. Therefore, GNP from *Gelidium crinale* can be used as a good potential active material for pharmaceuticals.

## Conclusion

In conclusion, a sulfated polysaccharide was successfully extracted from the red seaweed *Gelidium crinale*, which has a smaller molecular weight and a higher sulfate group. It has antioxidant effects and inhibits the activation of NF-κB and MAPK signaling in RAW 264.7 cells induced by LPS. GNP can effectively downregulate the production of inflammatory factors, which has strong anti-inflammatory properties. Our results provide experimental data for the further effective development and utilization of GNP and *Gelidium crinale*.

## Data Availability

The original contributions presented in the study are included in the article/Supplementary Material; further inquiries can be directed to the corresponding authors.
